# Comment on Hernandez-Vasquez et al., a bibliometric analysis of the global research on biosimilars

**DOI:** 10.1186/s40545-018-0144-z

**Published:** 2018-07-16

**Authors:** Hillel P. Cohen

**Affiliations:** Sandoz Inc., 100 College Road West, Princeton, NJ 08540 USA

**Keywords:** Bibliometric analysis, Biosimilar, Sandoz, Novartis

## Abstract

A bibliometric analysis provided by Hernandez-Vasquez et al. listed the institutions that have published most extensively in the field of biosimilars. Sandoz and Novartis International were considered as separate entities, but are both are divisions of the same parent company. When considered as a single entity for purposes of tracking publications, Sandoz-Novartis is among the leaders in the number of articles published about biosimilars.

Dear Editor,

The bibliometric analysis provided by Hernandez-Vasquez et al. [1] fills a gap in our knowledge of the growing literature in the field of biosimilars. One of the analyses conducted by the authors is identification of the top ten institutions publishing in biosimilars (Table 4). In this listing, Sandoz International is listed fifth, and Novartis International is listed tenth. Unfortunately, the authors may not be aware that Sandoz International and Novartis International are both divisions of one company - Novartis AG. Sandoz/Novartis should therefore be viewed as a single institution as scientists from the different divisions of Novartis work together as a single fully integrated biosimilars team.

With this clarification, when one adds the 33 publications ascribed to Sandoz and the 19 publications ascribed to Novartis, the resulting 52 would place Sandoz/Novartis first, just ahead of Amgen.

Unfortunately, it is not clear from Hernandez-Vasquez et al. if each publication is scored only once, using the institutional affiliation of the first or corresponding author, or if a given publication could be scored more than once if multiple institutions are listed. Were the latter to be the case and if Sandoz and Novartis were both listed on a single publication, the combined Sandoz/Novartis score would be lower.

In addition, combination of Sandoz and Novartis would lead to nine line listings on Table 4 instead of the desired ten.

Since we do not have access to the underlying data, we look forward to the authors providing an updated Table 4.

Sandoz/Novartis has been the pioneer in biosimilars since the inception of the industry. We began developing biosimilars in the 1990s, leading to the very first European, Japanese and US biosimilar approvals [2–4]. It is therefore not surprising that Sandoz/Novartis is among the leaders in publications within this nascent field.

Hillel P. Cohen.
